# The Broad Neutralizing Antibody Responses after HIV-1 Superinfection Are Not Dominated by Antibodies Directed to Epitopes Common in Single Infection

**DOI:** 10.1371/journal.ppat.1004973

**Published:** 2015-07-09

**Authors:** Valerie Cortez, Bingjie Wang, Adam Dingens, Mitchell M. Chen, Keshet Ronen, Ivelin S. Georgiev, R. Scott McClelland, Julie Overbaugh

**Affiliations:** 1 Program in Molecular and Cellular Biology, University of Washington, Seattle, Washington, United States of America; 2 Human Biology Division, Fred Hutchinson Cancer Research Center, Seattle, Washington, United States of America; 3 Vaccine Research Center, National Institutes of Health, Bethesda, Maryland, United States of America; 4 Department of Medicine, University of Washington, Seattle, Washington, United States of America; Miller School of Medicine, UNITED STATES

## Abstract

HIV-1 vaccines designed to date have failed to elicit neutralizing antibodies (Nabs) that are capable of protecting against globally diverse HIV-1 subtypes. One relevant setting to study the development of a strong, cross-reactive Nab response is HIV-1 superinfection (SI), defined as sequential infections from different source partners. SI has previously been shown to lead to a broader and more potent Nab response when compared to single infection, but it is unclear whether SI also impacts epitope specificity and if the epitopes targeted after SI differ from those targeted after single infection. Here the post-SI Nab responses were examined from 21 Kenyan women collectively exposed to subtypes A, C, and D and superinfected after a median time of ~1.07 years following initial infection. Plasma samples chosen for analysis were collected at a median time point ~2.72 years post-SI. Because previous studies of singly infected populations with broad and potent Nab responses have shown that the majority of their neutralizing activity can be mapped to 4 main epitopes on the HIV-1 Envelope, we focused on these targets, which include the CD4-binding site, a V1/V2 glycan, the N332 supersite in V3, and the membrane proximal external region of gp41. Using standard epitope mapping techniques that were applied to the previous cohorts, the present study demonstrates that SI did not induce a dominant Nab response to any one of these epitopes in the 21 women. Computational sera delineation analyses also suggested that 20 of the 21 superinfected women’s Nab responses could not be ascribed a single specificity with high confidence. These data are consistent with a model in which SI with diverse subtypes promotes the development of a broad polyclonal Nab response, and thus would provide support for vaccine designs using multivalent HIV immunogens to elicit a diverse repertoire of Nabs.

## Author Summary

Learning how to elicit a potent, cross-reactive neutralizing antibody (Nab) response capable of protecting against globally diverse human immunodeficiency virus-1 (HIV-1) subtypes is critical to the development of an HIV-1 vaccine. We and others have previously shown that HIV-1 superinfection (SI), or sequential infections from different partners, broadens and strengthens the Nab response. However, until now it was unclear whether SI also impacts the specificity, or epitope targets, of the antibody response. Previous studies have shown that the majority of singly infected individuals with broad and potent responses develop Nabs to 4 main epitopes on the HIV-1 Envelope. In contrast, here we show that none of the 21 SI cases in our Kenyan cohort developed Nabs that strongly target these epitopes. Our study helps to inform vaccine design by highlighting the prospect of eliciting broad and diverse HIV-specific Nab responses through sequential exposure to different HIV antigens.

## Introduction

Developing a neutralizing antibody (Nab)-based vaccine that is protective against diverse human immunodeficiency virus-1 (HIV-1) subtypes remains a major global health challenge [1]. While a number of different immunogens have been tested in both animals and humans [2], it is unclear which specific epitopes on the HIV-1 Envelope should be targeted by a vaccine and if vaccine-elicited Nabs to these epitopes will mediate protection [3]. To gain insight to this question, numerous studies have analyzed the epitopes that are targeted during natural HIV-1 infection [4–13]. Isolation of monoclonal antibodies (Mabs) from HIV-infected individuals, has identified four main epitope targets—the membrane proximal external region (MPER) in gp41, targeted by Mabs such as 10E8 [13], 4E10 [14], and 2F5 [15], the CD4-binding site, targeted by Mabs such as VRC01 [16], NIH45-46W [17,18], and HJ16 [19], glycopeptide residues in the V1/V2 region and also the V3 loop, targeted by Mabs such as PG9 [20] and PGT128 [21], respectively. Three previous screens of singly infected individuals with exceptionally broad and potent neutralizing activity have shown that typically 1–2 of these 4 main epitopes are the primary targets of their Nab responses [6,8,9]. Depending on the cohort, MPER and CD4-binding site-specific Nab responses were either not detected or were found to mediate up to a third of the cohort’s breadth, while glycan epitopes in the V1/V2 and V3 loops were each found to be targeted by 25–30% of individuals [6,8,9]. However, a small subset (1 or 2 individuals in each study) developed Nabs that could not be mapped to any of these 4 target regions [6,8,9], suggesting that there are additional epitopes that mediate a broad and potent Nab response. This idea is also supported by the recent identification of new epitopes, including 2 on the HIV-1 Envelope gp120-gp41 interface [22–25].

We and others have shown that superinfection (SI) leads to a broader and more potent Nab response when compared to single infection [26,27]. The observed difference in magnitude of the Nab response following SI is presumably due to increased antigenic stimulation from two viruses compared to one. However, it is not known whether SI also impacts Nab specificity. To determine whether SI stimulates the development of the same antibody specificities targeted by singly infected individuals with broad and potent responses, plasma samples were analyzed from 21 superinfected women from a cohort infected with HIV-1 subtypes A, C, and D. Using a combination of standard epitope mapping and computational analyses, we could not detect the presence of a dominant Nab response to the 4 main HIV-1 Envelope epitopes in any of the women’s plasma. Similar data were observed when plasma was tested for 4 additional epitope targets, 2 of which were those identified by isolation of new Mabs that target the gp120-gp41 interface [23,25]. This study, which is the first to examine the Nab specificities in a cohort of superinfected individuals, suggests that exposure to diverse HIV-1 antigens following SI may drive a broad and potent Nab response that may be mediated by polyclonal antibodies targeting multiple different epitopes.

## Results

### Broad and potent Nab responses in newly identified cases of SI

**Fig 1:**
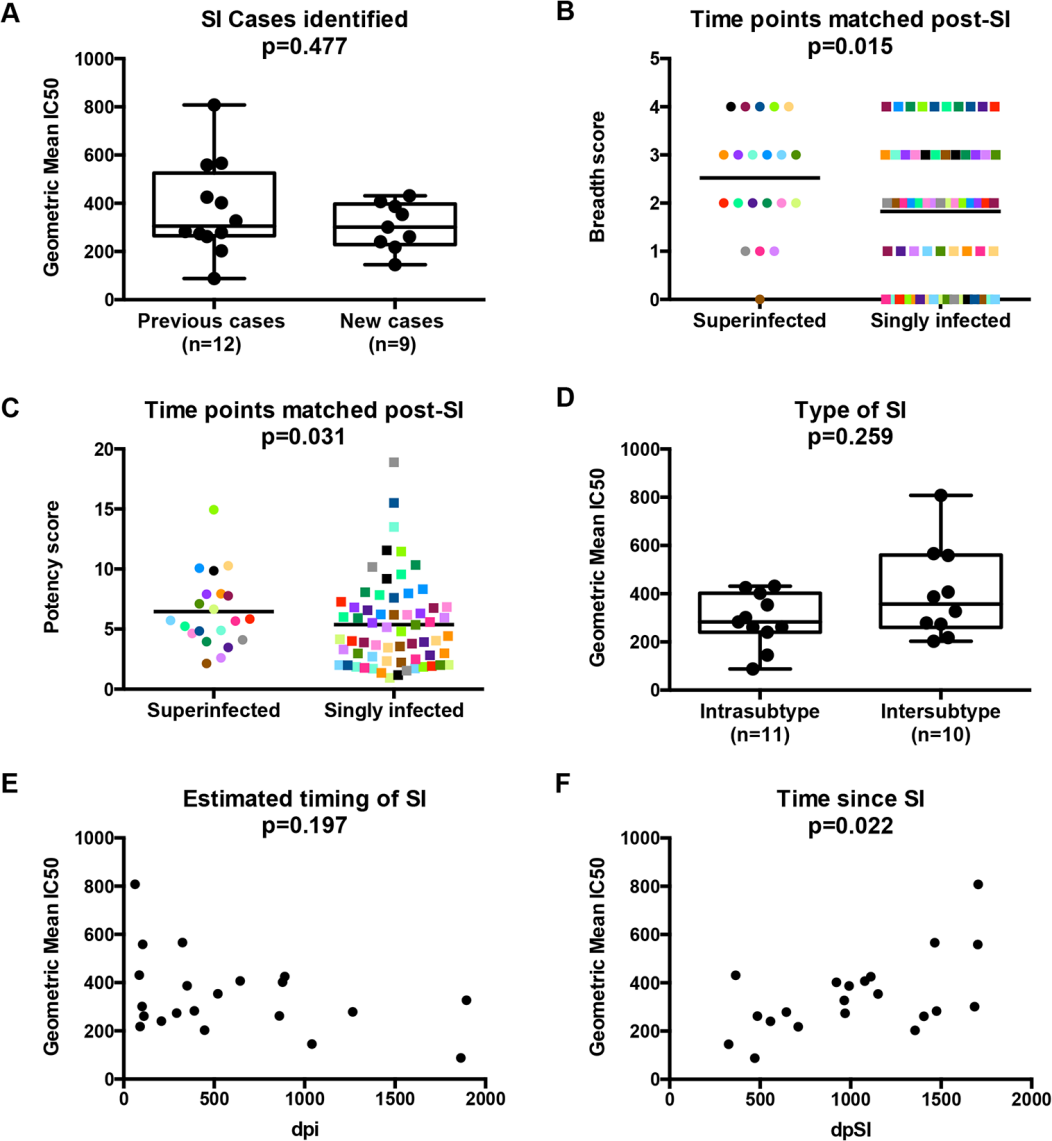
The relationships between SI and Nab breadth and potency. Comparison of Nab breadth between previously identified and new cases of SI is shown (A). Differences between Nab breadth (B) and potency (C) are displayed for the 21 cases of SI and 63 infected controls, with each matched group shown in the same color. Comparison of Nab breadth between individuals classified by type of SI is shown (D). The relationship of Nab breadth and (C) the estimated timing of SI, calculated as days post-initial infection, and (D) the time since SI, are shown on the bottom. In panels A and D-F, Nab breadth is summarized as the geometric mean IC50 obtained from testing ~5 year post-initial infection plasma from 21 superinfected women against an 8-virus cross-clade panel as defined in [27]. Mann-Whitney U tests were used for two group comparisons within the SI cohort, while Poisson regression and GEE were used to compare Nab responses between SI cases and singly infected controls. Spearman rank correlations were used for comparing continuous variables. P-values are indicated above each figure. dpi, days post-initial infection; dpSI, days post-SI.

The 21 cases of SI studied here consist of an original group of 12 that were identified by Sanger sequencing [28–30] and an additional 9 cases that were recently identified by 454 deep sequencing [31]. Combining all 21 cases, the timing of SI ranged from 63 to 1,895 days post-initial infection (dpi) (Table 1). While the broad and potent Nab responses of the original 12 cases have been previously described [27], the Nab responses of the additional 9 cases had not been characterized. To this end, plasma samples from the 9 cases obtained ~5 years post-initial infection were tested against the same cross-subtype panel of 8 viruses representing 4 different HIV-1 subtypes used in the previous study [27]. Six of the viruses were classified as more resistant to neutralization ‘Tier 2’ variants (Q769.b9, Q259.d2.26, Q842.d16, QD435.100M.a4, QC406.70M.f3 and DU156.12) and two viruses SF162 and Q461d1 were neutralization sensitive variants with Tier 1A and 1B classifications, respectively [27]. The ~5 years post-initial infection time point was studied here to capture a time in which all 21 cases had been superinfected for at least 1 year. The time point under study ranged from 365 days post-SI (dpSI) to 1,705 dpSI (median = 991 dpSI). Since 3 of the 9 new cases did not have a ~5 year time point available, plasma was tested at the last time point collected, as noted in Table 1. There was a broad range of neutralization activity observed across the 9 new cases of SI, with IC50s ranging from 100 to 3200 (S1 Fig). There was no significant difference in the Nab responses between the newly identified cases and the previously examined 12 when using the geometric mean IC50 across the virus panel as a summary measure for neutralization activity (p = 0.477, Mann-Whitney), demonstrating that these new cases have similarly broad and potent Nab responses (Fig 1A).

**Table 1:** Superinfection cohort (n = 21), sorted by geometric mean IC50 against 8-virus cross-clade panel.

ID	Estimated window of SI (dpi)	Estimated timing of SI (dpi)*	Type of SI^#^	Initial subtype	SI subtype	Time point studied (dpi)	Time point studied (dpSI)	Geometric mean IC50
**QB850**	52–73	63	Inter^^^	A	D	1768	1705	808
**QA013**	264–385	325	Inter^^^	D	A	1790	1465	566
**QC885**	58–152	105	Inter	A/C∞	A	1808	1703	559
**QC369**	29–143	86	Intra	A	A	451	365	451
**QB726**	749–1031	890	Intra	A/D	A	2002	1112	425
**QF564**	17–1270	644	Inter	A	D	1722	1078	407
**QB685**	303–1453	878	Intra	A	A	1800	922	402
**QF441**	255–444	350	Inter	A	D	1341	991	387
**QD151**	241–801	521	Intra	A	A	1701	1180	354
**QD022**	1832–1957	1895	Inter^^^	A	C	2859	964	327
**QG262**	59–144	102	Intra	A	A	1787	1685	301
**QC858**	341–440	391	Intra	A	A	1866	1475	283
**QA252**	1046–1487	1267	Inter	D	A	1912	645	279
**QB609**	101–485	293	Inter^^^	D	A	1262	969	274
**QA413**	714–1007	861	Intra	A	A	1346	485	262
**QD696**	49–174	112	Intra	A	A	1517	1405	261
**QG284**	155–260	208	Intra	A	A	765	557	240
**QB210**	17–63	90	Inter	A/D∞	C	800	710	218
**QB008**	303–591	397	Inter^^^	C	A	1803	1406	203
**QD149**	996–1086	1041	Intra	A	A	1367	326	145
**QB045**	1680–2048	1864	Intra	A/D	A	2334	470	88

*Midpoint between estimated window

^#^Classified as intersubtype if the strains belonged to different subtypes in at least one genomic region (*gag*, *pol*, *env*)

∞Two different subtypes found in different genomic regions

^Initial and superinfecting Envelopes are of different subtypes

dpi, days post-initial infection; dpSI, days post-superinfection

To further explore whether SI was associated with greater breadth of the Nab response among this group of 21 superinfected women, we compared the breadth and potency of the Nab response against a smaller virus panel (Q461d1, QD435.100M.a4, Q842.d16 and DU156.12) for these 21 cases and 63 matched singly infected controls. The 4 viruses were chosen based on the fact that this subset predicted the same outcome of the larger 8-virus screen among the first 12 SI cases and 36 controls [27], namely that SI is associated with a broader Nab response than single infection (Poisson regression; RR = 1.65, 95% CI: 1.08–2.50, p = 0.02). The same association was observed when the data from the original 12 cases was combined with the data from the 9 new cases and 63 singly infected controls (Poisson regression, RR = 1.38, 95% CI: 1.07–1.79, p = 0.015; Fig 1B), and remained significant after adjusting for viral load and CD4+ T cell count (RR = 1.42, 95% CI: 1.11–1.83, p = 0.006). The Nab response was also more potent in the women who were superinfected compared to those who were not (GEE, RR = 1.41, 95% CI: 1.01–1.29, p = 0.031; Fig 1C) and the point estimate was unchanged in the multivariate analysis, although with borderline significance (RR = 1.14, 95% CI: 0.99–1.31, p = 0.06).

### Exploratory analysis reveals time since SI is a correlate of Nab breadth

An exploratory analysis was performed to determine if particular factors related to SI were predictive of Nab breadth. Ten of the 21 women were cases of intersubtype SI based on the detection of subtype differences in at least one HIV-1 genomic region (*gag*, *pol*, or *env*), and the other 11 women were defined as cases of intrasubtype SI [28–31] (Table 1). These data are limited by the fact that the genomic regions sampled were larger in the first 12 SI cases versus the second group of 9 cases due to differences in sequencing methods (Sanger versus 454). In particular, the 454 sequencing captured an approximately 500 bp region spanning the C2-V3 of *env*, which most likely does not capture the collection of relevant epitopes that could drive Nab breadth, as suggested by a recent study [32]. Using these data, we did not observe a statistically significant association between the type of SI and neutralizing activity (p = 0.259, Mann-Whitney, Fig 1D). However, it is notable that the top 3 cases with the highest geometric mean IC50s were all cases of intersubtype SI, whereas the 2 women with lowest geometric mean IC50s were cases of intrasubtype SI (QA149 and QB045) (Table 1). There was no statistically significant association between the estimated time of the SI event in relation to first infection and neutralizing activity at ~5 years post-initial infection (p = 0.197, Spearman’s rank correlation, Fig 1E); however, the same top 3 cases with the highest geometric mean IC50 had all become superinfected within the first two years following initial infection, while the 2 women with the lowest geometric mean IC50 became superinfected many years following initial infection (average: 3.98 years). These descriptive data hinted that the timing of SI could be playing a role, and indeed, the length of time since SI at the time of sampling and geometric mean IC50 were correlated (p = 0.022, Spearman’s rank correlation), suggesting that the longer an individual harbored two viruses, the stronger the Nab response developed during chronic infection (Fig 1F). Likewise, previous studies of singly infected populations have shown that duration of infection is correlated with Nab breadth [6,33–35].

### Infrequent detection of MPER-specific antibodies

**Fig 2:**
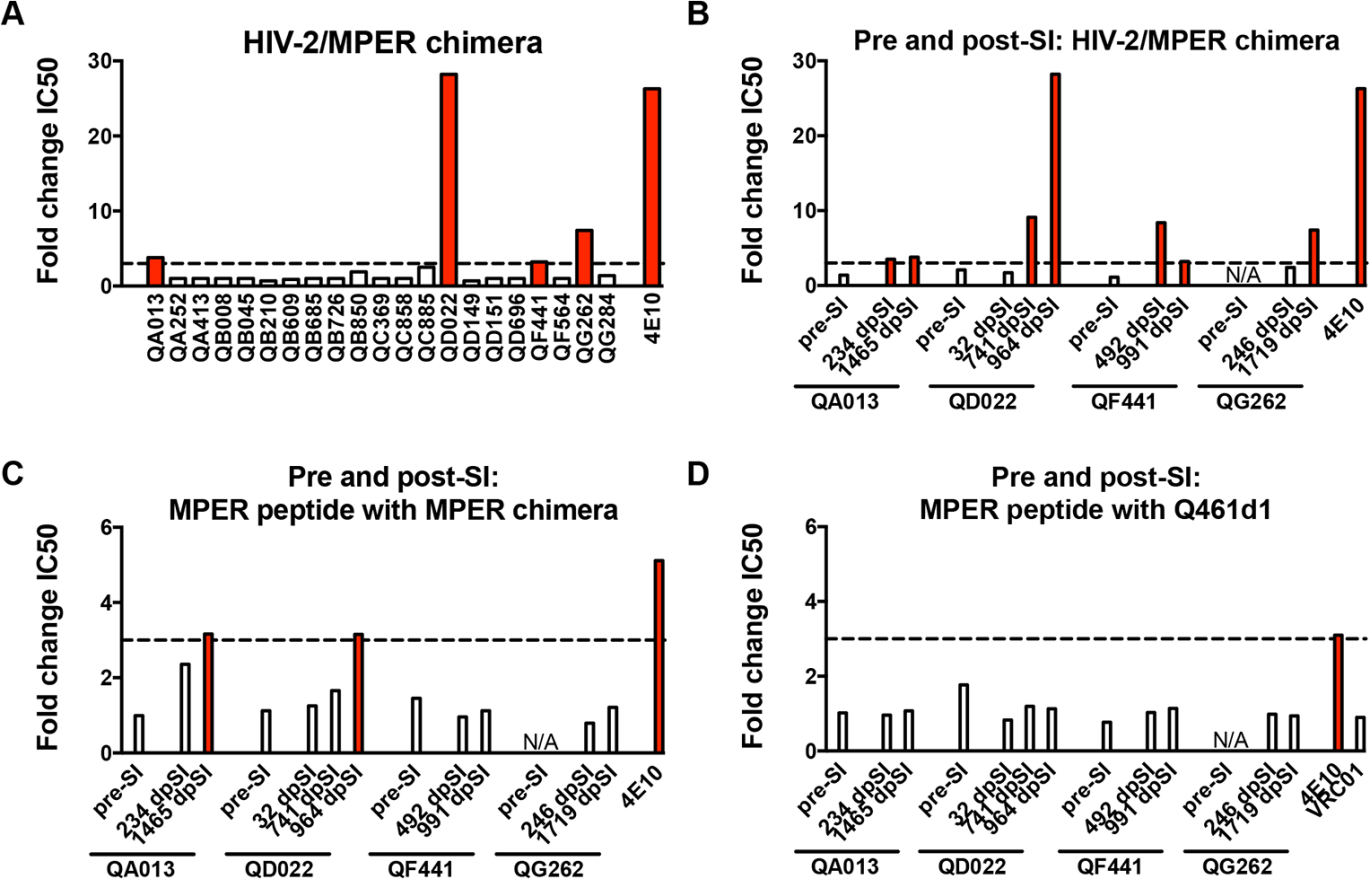
Neutralization of HIV-2 and an HIV-2/HIV-1 MPER chimera and competition with MPER peptides indicate MPER reactivity in plasma. Fold change in IC50 (HIV-2:MPER chimera) for all 21 superinfected women (A) and longitudinal plasma pre- and post-SI from 4 superinfected cases with MPER reactivity ~5 years post-initial infection (B). Fold change in neutralization of the MPER chimera (C) or HIV-1 subtype A virus Q461d1 (D) in the absence or presence of MPER peptide. In all panels, >3-fold changes in IC50 are noted with red bars. 4E10, an MPER-specific Mab, is shown in all panels as a positive control and VRC01, a CD4-binding site-specific Mab, is shown as a negative control in panel D. N/A denotes a time point not tested in these experiments. dpSI, days post-SI.

Neutralization of an HIV-2/HIV-1 MPER chimeric virus (7312C1) was used as an initial screen for MPER-specific antibodies in post-SI plasma from all 21 women. Neutralization of the MPER chimera at a level >3-fold than what is observed for HIV-2 (7312A) indicates the potential presence of MPER-specific antibodies [13]. Four of the 21 superinfected women (QA013, QD022, QF441, QG262) demonstrated MPER reactivity using this assay, with changes in IC50 between HIV-2 and the MPER chimera ranging between 3.2 and 28.2 (Fig 2A). To determine when these responses arose in these 4 individuals, plasma samples were tested at time points pre- and post-SI, except for QG262, who was only tested post-SI because this individual was superinfected early after initial infection (102 dpi), before Nabs typically develop [36] (Fig 2B). None of the 3 individuals tested had detectable MPER-specific Nab responses pre-SI. Following SI, QA013 and QF441 showed 3.5–fold and 8.4–fold changes, respectively, in IC50 between HIV-2 and the MPER chimera (234 dpSI and 429 dpSI, respectively). Neither of the other two individuals, QD022 and QG262, demonstrated evidence of MPER reactivity immediately following SI (32 dpSI and 246 dpSI, respectively). However, QD022 demonstrated a 9.1-fold change in IC50 between HIV-2 and the MPER chimera ~2 years after SI (742 dpSI), and this MPER activity later peaked after 2.6 years after SI (964 dpSI, 28.2-fold change). QG262 did not demonstrate strong MPER reactivity until ~4.7 years after SI (1719 dpSI, 7.4-fold change). Together these data suggest that the MPER-specific responses measured by this assay occurred *de novo* post-SI, but 3 of the 4 individuals did not develop these responses until >1 year following SI.

While HIV-2/HIV-1 MPER chimera neutralization has been widely used as a rapid screen for MPER reactivity, blocking experiments using MPER peptides provide another measure for MPER-specific activity [7,9,10,13]. To better define whether MPER-specific antibodies mediate the HIV-specific neutralizing activity of these women’s plasma, MPER peptides matching the sequence of the chimera were used to absorb MPER-specific antibodies prior to a neutralization assay using the chimera and HIV-1 viruses. Fig 2C illustrates that MPER peptides did not have a large effect (average: <1.30-fold reduction) on the neutralization of the MPER chimera for any of the four individuals at time points pre-SI or immediately post-SI, or for QF441 and QG262 at ~5 years post-initial infection. There was some evidence of MPER activity for both ~5 year post-initial infection plasma from QA013 and QD022, as the addition of MPER peptides resulted in 3.16 and 3.15-fold reductions in IC50, respectively. However, none of the plasma samples from the 4 individuals at time points pre or post-SI demonstrated >3-fold reductions in IC50 against the HIV-1 variant, Q461d1, after the addition of MPER peptides (Fig 2D). As expected, neutralization of the MPER chimera and Q461d1 by MPER-specific Mabs 4E10 and 10E8 was substantially decreased with the addition of MPER peptides (average: >4-fold reduction), while VRC01 neutralization of Q461d1 was unaffected (average: 0.90-fold change) (Fig 2D). Similar results were also observed using the subtype C virus, DU156 (S2 Fig). Since neutralization was unaffected in these 4 individuals’ plasma when tested against HIV-1 viruses, we conclude that it is unlikely that MPER-specific Nabs mediate a substantial fraction of their neutralizing activity, although we cannot rule out that they may contribute to a polyclonal response.

### CD4-binding site-specific antibodies are rare and not a target for neutralization

**Fig 3:**
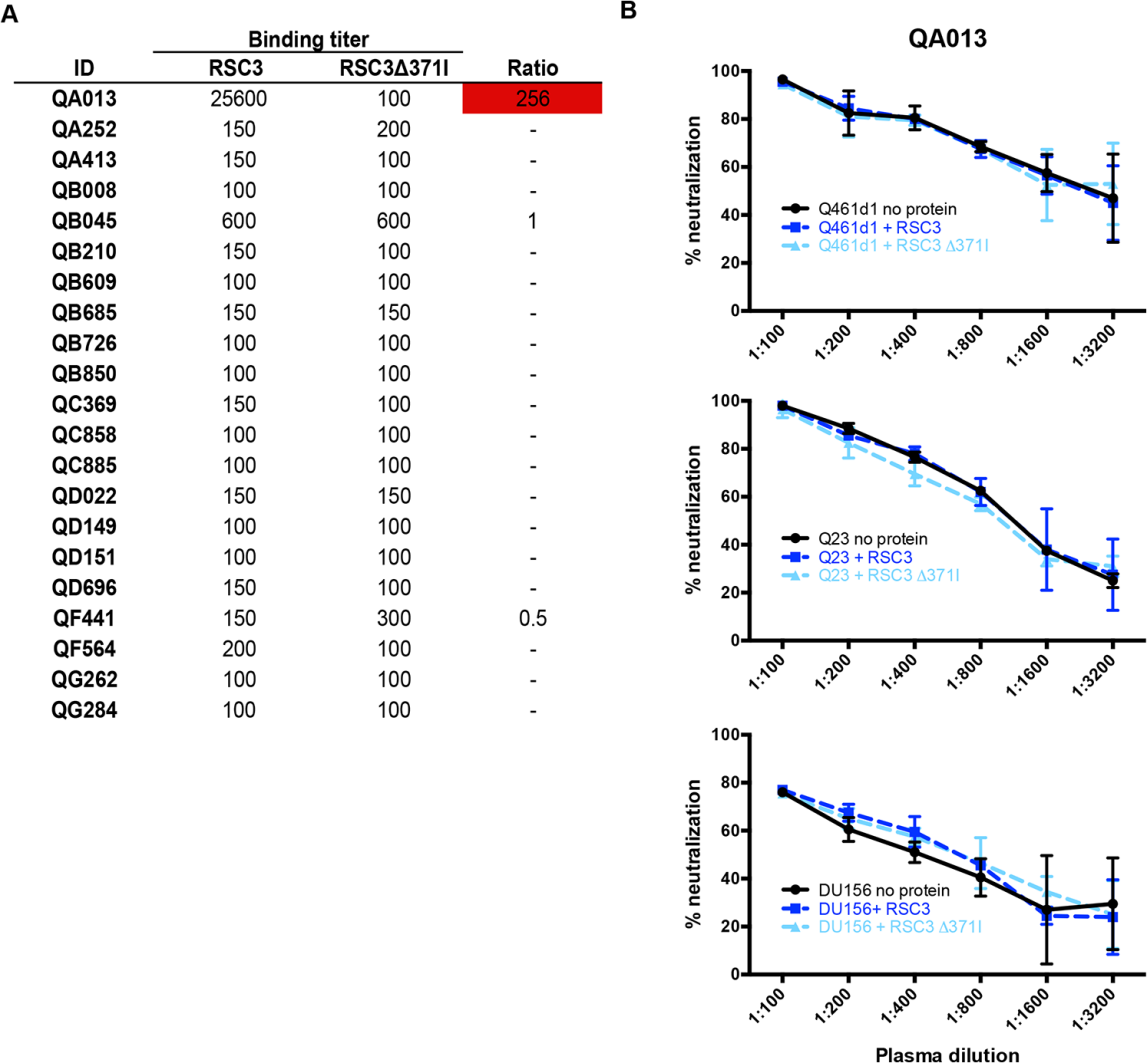
Differential binding of RSC3 and RSC3Δ371I proteins and their ability to compete for CD4-binding site-specific antibodies in a neutralization assay. (A) Post-SI plasma from all 21 superinfected women were tested for binding to RSC3 wildtype and RSC3Δ371I mutant gp120. Ratios (wildtype:mutant) of endpoint titers are shown in the far right column, with ratios >3 noted in red. Ratio was not calculated (-) when reciprocal endpoint titers were both <200. (B) QA013 ~5 years post-initial infection plasma tested against Q461d1, Q23, or DU156 alone (black) with the addition of RSC3 wildtype (dark blue) or mutant proteins (light blue).

Detection of CD4-binding site-specific antibodies in plasma was performed by comparing binding affinity to the resurfaced-core 3 (RSC3) gp120 protein, which has been engineered to prominently display the CD4-binding site, and the mutant protein, RSC3∆371I, which contains a single deletion that abrogates binding of most CD4-binding site-specific antibodies [5,16]. This assay detects both neutralizing and non-neutralizing antibodies directed to the CD4-binding site. Only 1 of the 21 women (QA013) developed antibodies that bound RSC3 (Endpoint titer: 1:256,000) stronger than the RSC3∆371I mutant (Endpoint titer: 1:100). The plasma from the other 20 women was either non-reactive to either protein (n = 18; endpoint titer <1:200) or did not bind the 2 proteins differentially (n = 2; ratio <3-fold) (Fig 3A).

Next, to determine whether QA013’s CD4-binding site-specific antibodies were capable of neutralizing HIV-1, RSC3 proteins were used to compete for binding of these antibodies in a standard neutralization assay. Addition of the RSC3, but not the RSC3∆371I protein, resulted in a 5 to 10-fold reduction in neutralization for the CD4-binding site-specific Mab, VRC01, when tested against two subtype A viruses, Q416d1 and Q23, as well as one subtype C virus, DU156 (S3 Fig). Neutralization by PG9, a V1/V2 glycan specific Mab, was unaffected by either RSC3 proteins (S3 Fig). Likewise, neither RSC3 nor RSC3∆371I proteins altered the neutralization of Q461d1 by QA013 plasma (Fig 3B). Similar results were also seen with Q23 and DU156 viruses (Fig 3B), demonstrating that QA013 developed binding, but not neutralizing CD4-binding site-specific antibodies following SI.

### No strong evidence of V1/V2 glycan-specific Nabs

**Fig 4:**
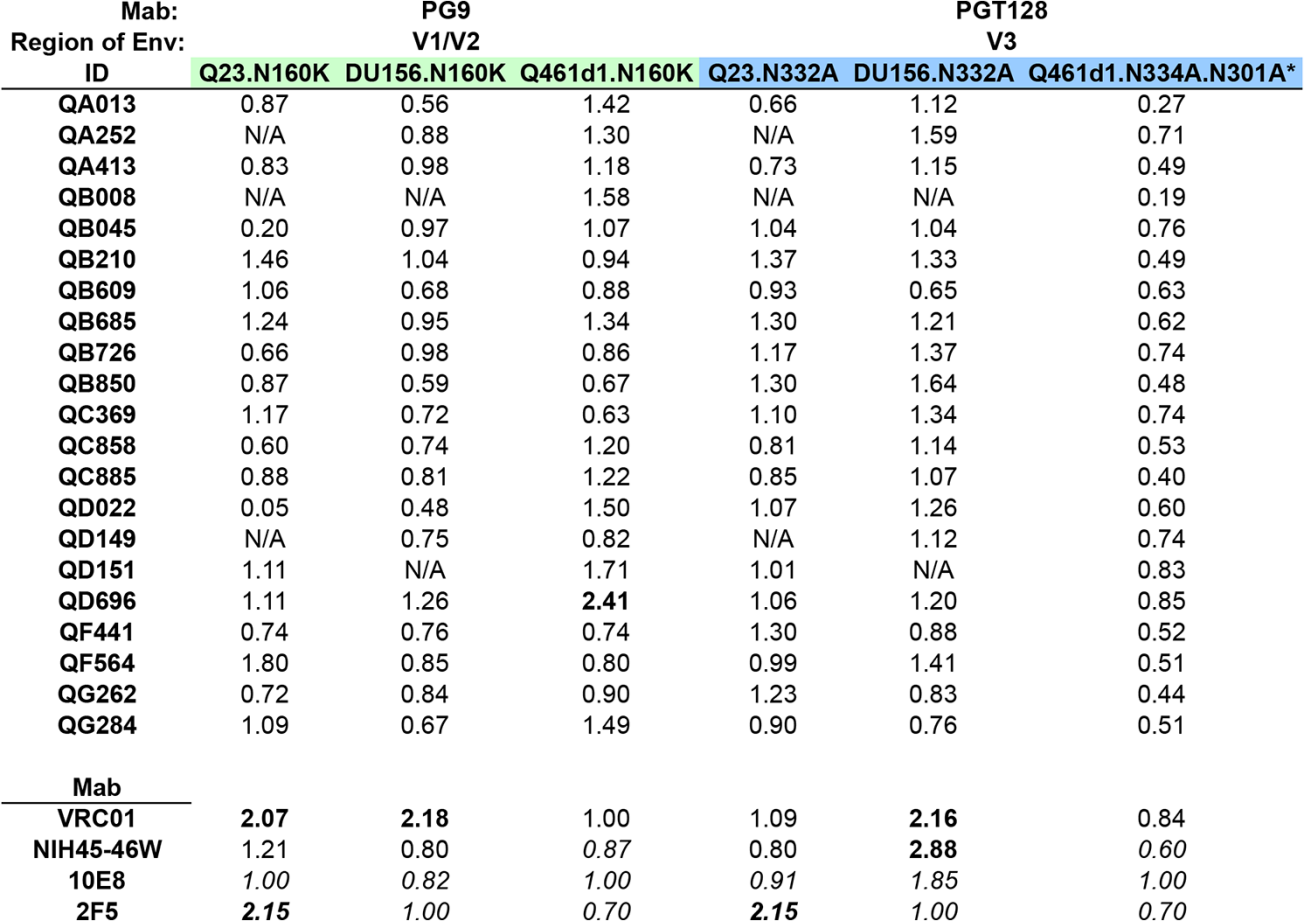
Inhibition of neutralization by point mutations that disrupt V1/V2 and V3 glycan-specific Nab recognition. Fold change in IC50 (wildtype:mutant) for each set of virus pairs are shown for all 21 cases of SI, with colors denoting the different mutated residues, corresponding to specific epitopes targeted by Mabs listed above each panel. Fold changes in IC50 are shown for N160K and N332A/N334A/N301A mutations. Mab controls, including VRC01 and NIH45-46W, both CD4-binding site-specific Mabs, and 2F5 and 10E8, both MPER-specific Mabs are shown below. Fold changes >2 are highlighted in bolded font. *Q461d1 WT virus not neutralized by PGT128 (20 ug/ml). IC50s in italics denote data from duplicate testing within a single experiment. N/A denotes plasma samples that were unable to neutralize the wildtype virus at the plasma dilutions tested.

The PG class of Mabs largely requires the presence of an N-linked glycan at position 160 in the V1/V2 region of Envelope for potent neutralization [20]. As predicted, removal of this glycan abrogated neutralization of Q461d1 (subtype A), Q23 (subtype A) and DU156 (subtype C) viruses by PG9 >32-fold reduction in IC50, enabling their use to assess PG-like neutralizing activity in plasma. To next define the potential for non-specific effects resulting from loss of the N160 glycan, wildtype (WT) and N160K mutant viruses were tested against the CD4-binding site-specific Mab, VRC01. The N160K mutation caused a decrease in VRC01’s ability to neutralize 2 of the 3 viruses (Q23: 2.07-fold change, DU156: 2.18-fold change) (Fig 4). There were also some modest non-specific effects observed with other Mabs that also do not target the glycan at N160 (2F5, 10E8, NIH45-46W, Fig 4), indicating that the removal of this glycan may result in conformational changes of the Envelope trimer that impact recognition by Nabs that do not include this amino acid within their epitope. Although this non-specific effect was modest relative to the effect of these mutations on PG9 neutralization, in order to account for it, a 3-fold change in IC50 was used as a cutoff for plasma samples to confirm the presence of N160 glycan-specific Nabs.

None of the 21 plasma samples demonstrated greater than a 3-fold change in IC50 against Q461d1, Q23, nor DU156 (Fig 4). Some individuals demonstrated less potent neutralization against the test viruses, and thus made distinguishing a >3-fold change challenging if their IC50 against the WT was <300 (S4 Fig). We note that for these cases, the neutralization curves were very similar for WT and mutant viruses and thus supported a lack of change in neutralization between the WT and mutant. However, we found 1 individual (QD696) who showed a >2-fold change in IC50 with Q461d1, but this was not consistent with the results from the other two viruses tested, Q23 and DU156 (Fig 4). Given that it did not reach the 3-fold threshold and was not seen across viruses, we interpret these findings to suggest that PGT nabs did not constitute a significant fraction of this individual’s response.

### No strong evidence of V3 glycan-specific Nabs

Removal of the N332 glycan has been shown to disrupt neutralization of the PGT class of Mabs, including PGT128 [21,37,38]. Consistent with this, introduction of an alanine at this site to remove this glycan from Q23 and DU156 abolished neutralization by PGT128 (14-32-fold reduction in IC50). To assess the potential for non-specific effects resulting from the removal of these glycans, the WT and mutant Envelopes were tested against Mabs that do not target this epitope, including VRC01, 2F5, 10E8, and NIH45-46W. When testing VRC01, the introduction of N332A in the 2 test viruses resulted in a 2.16-fold change in IC50 for DU156 and little change in neutralization for Q23. Larger non-specific effects were observed with 10E8 (1.85-fold change, DU156), NIH45-46W (2.88-fold change, DU156), and 2F5 (2.15-fold change, Q23) (Fig 4). To account for these non-specific effects, a greater than 3-fold change in IC50 was required to indicate a strong presence of V3 glycan-specific antibodies.

None of the 21 individuals’ plasma samples demonstrated greater than 3-fold or even 2-fold changes in IC50 between any of the sets of WT and mutant viruses (Fig 4). Again, while some plasmas samples showed neutralization below a 1:300 dilution against the WT virus (S4 Fig), examination of the neutralization curves between the WT and mutant showed that they were not notably different. As a means to address this issue, we also performed experiments with a more neutralization sensitive virus, Q461d1. Although we found that PGT128 was unable to neutralize the WT Q461d1 at a starting concentration as high as 20 ug/ml, we chose to use this Envelope because it is potently neutralized by the majority of individuals in the SI cohort. Q461d1 lacks residue N332, but instead has shifted its asparagine residue to position N334 [38]. Thus, an alanine was introduced to Q461d1 at position N334, and as an additional measure, a second alanine was introduced at position N301, which can also be utilized by the PGT class of Mabs when the N332/N334 glycans are absent [38]. None of the Mabs that target epitopes outside these residues were largely affected by their removal (Fig 4). Similar to what was seen with Q23 and DU156, there was no evidence that these mutations disrupted recognition of the Nabs present in the 21 plasmas from SI cases (Fig 4). Thus, these data suggest that these women’s responses did not strongly target these V3 glycans following SI.

### Screening for Nabs targeting residues beyond the 4 dominant sites on HIV-1 Envelope

Additional epitopes beyond the 4 tested here and in prior screens of single HIV infections have been recently defined on the HIV-1 Envelope after the characterization of broadly neutralizing Mabs 8ANC195, PGT151, and 35O22 [23–25]. 8ANC195’s binding and neutralization involves the residue N276 in C2, while PGT151 and 35O22 target distinct epitopes at the gp120-gp41 interface, involving residues in the C-C loop in gp41 (N611) and C1 (N88), respectively. K169 in V1/V2 has also been shown to be critical for the neutralization of the recently characterized Mab CAP256.VRC26.08 [39], and was previously shown to be involved in binding by other V1/V2-targeting Mabs, such as PG9 [40]. While it is unclear how prevalent these types of antibodies are in HIV-infected populations, the fact that we could not find evidence for dominant Nab responses to the 4 main epitopes led us to assess whether the 21 SI cases developed antibodies to these additional targets. To this end, point mutations were engineered to disrupt the above epitopes in the subtype A Envelope, Q461d1. The majority of these mutations abrogated the neutralization activity of their cognate Mab, however, the N611A mutation caused a dramatic 33-fold enhancement of neutralization by PGT151 (0.03-fold change IC50), and the N88A virus could not be generated at high enough concentrations for testing in the neutralization assay. These residue changes were instead successfully engineered into the background of another subtype A Envelope, Q23, with a loss of neutralization noted by PGT151 for N611A and 35O22 for N88A (18-fold and 32-fold, respectively).

**Fig 5:**
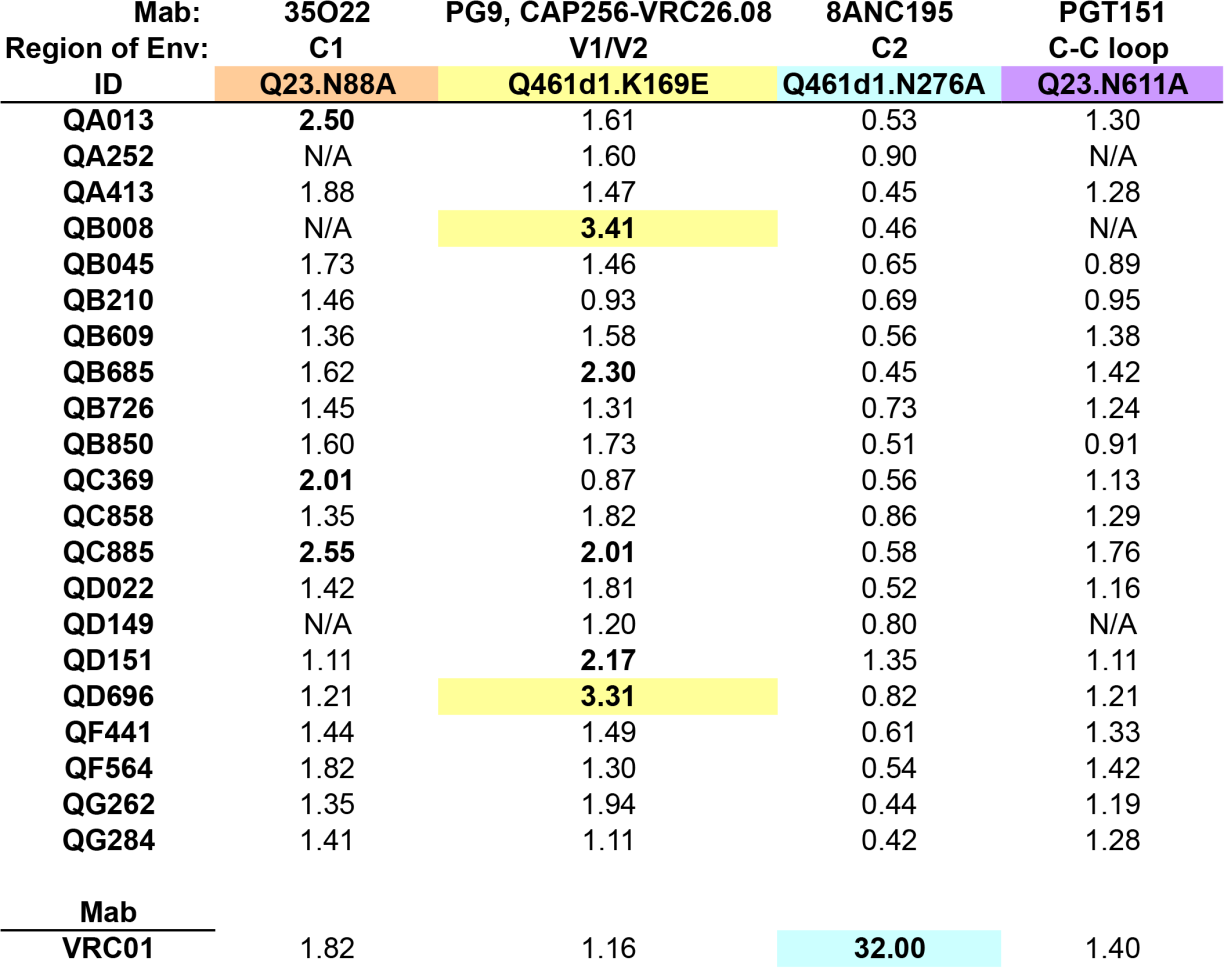
Inhibition of neutralization by point mutations that disrupt epitopes beyond the 4 main targets on Envelope. Fold change in IC50 (wildtype:mutant) for each set of virus pairs are shown for all 21 SI cases, with colors denoting the different mutated residues, corresponding to specific epitopes targeted by Mabs listed above each panel. Fold change in IC50 for VRC01, the CD4-binding site-specific Mab, is shown below. Fold changes >2 are highlighted in bolded font, >3 are additionally highlighted with colored backgrounds. N/A denotes plasma samples that were unable to neutralize the wildtype virus.

Initial testing of VRC01 against each WT and mutant pair enabled us to gauge the level of non-specific activity caused by the residue change. Neither K169E nor N611A mutations substantially altered the neutralization by VRC01, whereas N88A caused a 1.82-fold change in IC50 and N276A caused a considerable 32-fold change in IC50 (Fig 5). Interestingly, while VRC01 has been shown to interact with the N276 residue, it is not thought to be a critical component, as the loss of the glycan does not affect binding [41]. However, in some cases, mutation of this N276 residue can cause an enhancement of neutralization (0.07 to 0.31-fold change in IC50) [42], suggesting that VRC01’s dependency on this residue may be virus-specific. Testing these sets of viruses against plasma from all 21 women revealed that none were strongly affected by the N276A or N611A mutations (Fig 5). Plasma from only two individuals exhibited over 3-fold changes in IC50 (QB008: 3.41-fold; QD696: 3.31-fold) when tested against the Q461d1 K169E mutant; three individuals also showed a >2-fold change. Three different individuals also showed a >2-fold change in IC50 when tested against the Q23 N88A mutant, but none showed a 3-fold change or greater. Again, neutralization curves generated from plasma samples that did not potently neutralize the WT virus were closely examined and were found to be consistent with a lack of change in neutralization between the WT and mutant (S5 Fig). Together these data suggest that N88 and K169-targeted antibodies are not significant drivers of the Nab response in the majority of SI cases, but K169-directed antibodies could potentially represent a fraction of the response in 2 individuals.

### Longitudinal analysis of QB850 and QA013 plasma concordant with epitope mapping ~5 years post-initial infection

**Fig 6:**
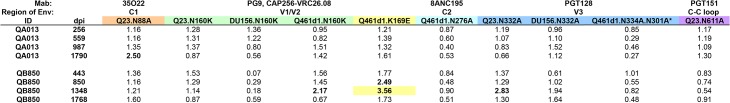
Longitudinal analysis of epitope targets in QB850 and QA013 plasma. Fold change in IC50 (wildtype:mutant) for each set of virus pairs are shown for all 21 SI cases, with colors denoting the different mutated residues, corresponding to specific epitopes targeted on Envelope by Mabs listed above each panel. Fold changes >2 are highlighted in bolded font, >3 are additionally highlighted with colored backgrounds. *Q461d1 WT virus not neutralized by PGT128. IC50s in italics note data is from a single experiment.

Since previous studies have shown that more than one Nab specificity can arise after years following infection [43–46], this may explain why ~5 year post-initial infection plasma from the 21 SI cases appear to lack a dominant Nab response. Thus, to investigate whether Nabs targeting these epitopes developed prior to this time point, longitudinal plasma samples from the two individuals with the broadest responses in the cohort, QB850 and QA013, were tested against WT and N88A, N160K, K169E, N332A/N334A/N301A, N276A, and N611A mutants in 1 to 3 different Envelope backgrounds as noted in Fig 6 and S6 Fig. Testing QA013 plasma obtained from a pre-SI time point at 256 dpi, and then post-SI at 559 and 987 dpi did not reveal any notable changes in neutralization capacity in the absence of these key residues. Post-SI plasma samples from QB850 at time points 443 dpi, 850 dpi, and 1348 dpi were next tested against the same panel of WT and mutant viruses. Overall, while no changes >3-fold were observed at the two earlier time points, a 3.56-fold change in IC50 was noted at 1348 dpi for the K169E mutation in Q461d1. These data suggest that at 1348 dpi, QB850 potentially developed a minor fraction of Nabs that targets a similar epitope as Mabs PG9 and CAP256.VRC26.08, however, this level of response was not sustained, as there was <2-fold change in IC50 noted at 1768 dpi. Thus, no single specificity appeared to dominate QB850 response at any of the longitudinal time points tested.

### Computational analysis and mapping with autologous Envelope clones shows evidence of PGT-like neutralizing activity in one SI case

To complement these functional assays, the ~5 year post-initial infection plasma samples for all 21 SI cases were next tested against a panel of 21 viruses to perform a neutralization fingerprinting analysis for serum delineation [47]. For 20 of the 21 SI cases, a definitive computational signal could not be identified (S7 Fig). The remaining individual, QB850, who also had the most potent response (Table 1) had a neutralization profile that demonstrated a relatively high signal (>0.5) for a PGT-like activity targeting V3 glycans.

Given that we did not detect evidence of PGT-like antibodies in our functional screen of QB850 ~5 year post-infection plasma, additional experiments using autologous Envelope clones were initiated to further investigate the presence of PGT-like Nabs. Three time points were chosen for cloning, starting with the first time point that SI was first detected—73 dpi, followed by two later time points—324 dpi and 632 dpi. Of the 9 functional Envelope clones isolated from 73 dpi, 3 were SI recombinants, whereas the remaining 6 clones were initial variants. All clones isolated from later time points were SI recombinants (S8A Fig).

All 15 autologous viruses were tested against longitudinal plasma samples collected from QB850 (S8B–S8D Fig). To initially investigate the epitope targets developed earlier in QB850, sequence alignments were compared between the 73 dpi A1 clone that was sensitive to 443 dpi plasma (IC50: 2251) and 324 dpi D2 that was resistant (IC50: <100). The alignment revealed a major difference in the C1-V2 region of 73p.A1, which is derived from the SI subtype D virus. Generation of a chimeric virus containing the C1-V2 of 73p.A1 inserted into the 324p.D2 clone did not render the clone sensitive to neutralization (IC50: <1:100), suggesting that the ‘site of vulnerability’ on the 73p.A1 clone lies outside of this region. Interestingly, the alignment of all 15 autologous sequences did not show evolution of N301 and N332 residues over time (S8A Fig), which is consistent with the findings from functional studies that these are not major targets of the Nab response.

**Fig 7:**
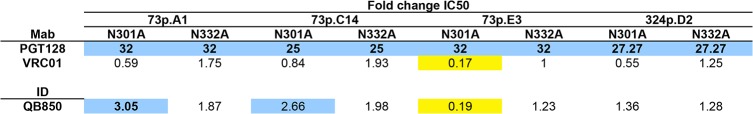
Inhibition of neutralization by QB850 autologous mutants that disrupt V3 glycan-specific Nab recognition. Fold changes in IC50 (wildtype:mutant) calculated after testing wildtype initial and superinfecting variants, alongside N301A and N332A mutants against PGT128, a V3 glycan-specific Nab, VRC01, a CD4-binding site-specific Nab, and QB850 ~5 year post-initial infection plasma (B). Fold changes >2 are highlighted in bold font, >3 are additionally highlighted with blue backgrounds, and <0.20, denoting neutralization enhancement, are shown in yellow. dpi, days post-initial infection.

To further investigate whether there was evidence for PGT-like Nabs in QB850 ~5yr plasma as predicted by the computational analysis, 3 clones isolated from the 73 dpi time point and 1 clone from 324 dpi were chosen to generate N332A and N301A mutations. Both mutations abrogated neutralization by PGT128 when engineered into these 4 Envelope clones (Fig 7). There was little difference between VRC01’s ability to neutralize the WT as compared to the mutant viruses (<2-fold) (Fig 7). However, introduction of the N301A mutation into 73p.E3 resulted in the enhancement of VRC01 neutralization in comparison to the wildtype (0.17-fold change) (Fig 7). When the 8 glycan mutants were tested against autologous post-SI plasma (1,705 dpSI), only 1 of the 8 resulted in reduction in neutralization above background (3.05-fold change), while the N301A mutation in 73p.E3 resulted in a 5-fold enhancement of neutralization (0.19-fold change) similar to what was observed with VRC01 (Fig 7). These data could suggest that the loss of the N301 glycan in this E3 clone causes a global change in Envelope conformation. Overall, the N332A and N301A mutations reduced QB850 plasma neutralization in only 1 of the 8 autologous clones tested, with fold changes in neutralization not much different than what was seen with Mabs that do not target these epitopes and ~10 fold less than the effects on Mabs that do recognize this epitope. These relatively small changes make it difficult to determine if these are simply non-specific changes or indicative of a small contribution of PGT-like Nabs to QB850’s response. Overall, we interpret these data to suggest that since neutralization was not completely abrogated upon introducing these mutations, V3 glycan-specific Nabs may have constituted at best a minority fraction of QB850’s overall Nab response.

## Discussion

Designing immunogens to elicit a broad and potent Nab response that protects against diverse HIV-1 variants remains a critical endeavor to achieving an AIDS-free generation [48]. The most recent advances in this area of study have stemmed from isolating and characterizing broad Nabs from HIV-infected individuals, with their ontogeny and the epitopes they target potentially serving as a guide for future vaccine design [49]. We and others have reported that superinfected individuals develop broad and potent Nab responses, and therefore may provide additional mechanistic insight to how these types of responses develop [26,27]. The analysis of an additional 9 cases of SI and the aggregate data from the 21 cases of SI identified in the Mombasa cohort presented here supports these earlier findings. While multiple studies have addressed the epitope targets associated with broad Nab responses in single infection [4–13], this had not been previously examined in a cohort of superinfected individuals. Indeed, several studies have shown that 72–94% of HIV-1 singly infected individuals with broad and potent responses develop antibodies that can be ascribed to targeting 1 of the 4 main epitopes examined here [6,8,9]. Conversely, we found that none of the SI cases in our Kenyan cohort developed Nab responses that were dominated by antibodies targeting these epitopes or 4 others that were more recently identified. Importantly, we applied similar methods for probing antibody specificity that were used in prior studies so that the results from our screen could be placed in the context of those previously published [6,8,9]. These data suggest the possibility that polyclonal antibodies targeting multiple different epitopes mediate the broad and potent Nab responses following SI.

Our studies do not rule out the possibility that antibodies targeting these four sites of vulnerability do not contribute to the Nab responses in these cases of SI, as the assays employed are not ideal for detecting a specificity that comprises a minor portion of a polyclonal response. Overall, in a subset of SI cases we found evidence for at most a minority fraction of neutralizing activity directed to one of the 8 sites tested here. Neutralization assays using an HIV-2/HIV-1 MPER chimera suggested that 4 individuals (QA013, QD022, QG262, QF441) showed some MPER reactivity that arose following SI. However, closer examination with MPER peptides used in competition neutralization assays with HIV-1 variants did not alter neutralization. Probing for CD4-binding site-specific antibodies using a combination of ELISAs and neutralization assays identified one individual, QA013, who developed binding, but not Nabs to this epitope. Beyond the two individuals who showed some evidence of having antibodies that target the K169 residue, very little neutralization activity targeting glycans in V1/V2 or V3 as well as epitopes at positions N88, K169, N276, and N611, was observed. Furthermore, we found that these data were not consistent across Envelope backgrounds, were often on par with the level of non-specific effects observed in the assay, or not far above the 3-fold cutoff. Together, these functional data revealed that a subset of the 8 sites tested here may have developed in a few of the SI cases, but we could not find evidence that they individually significantly contributed to these individuals’ ability to neutralize diverse HIV-1 Envelopes.

One caveat to the data from the 21 women screened here is that we only tested a single time point to examine epitope specificities, which may have decreased our ability to detect a transient monoclonal response. There are several studies of singly infected individuals where different dominant specificities were detected at time points over multiple years of infection or where Mabs with different specificities have been isolated from a single time point [43–46]. To address this possibility, we focused on longitudinal plasma from the 2 women with the broadest responses, QB850 and QA013, but once again found no evidence of a dominant response to an epitope common to single infection. Together these data suggest that the Nab responses in these individuals starting as early as 1 year after SI until the time when considerable breadth has developed do not reflect the presence of a monoclonal response to a known epitope.

While we did not detect evidence for a dominant antibody response directed to these main epitopes in our Kenyan cohort of women exposed to multiple subtypes, this does not rule out the possibility that these responses can occur as a result of SI. In fact, there is a case report of an intrasubtype C superinfected individual where a robust V1/V2 antibody response developed [44]. However, since the subtype C superinfected case was identified after screening 40 individuals to detect only 7 individuals with Nab breadth, it is unclear if this is an atypical event in subtype C infections or common relative to the mixed subtype infections studied here. There is also another case of subtype C coinfection that led to a V3 glycan-specific response [50]. Subtype C infections are rare in our Kenyan cohort, and none of the cases here were the result of infection with two subtype C viruses. Rather, the individuals with the greatest breadth were superinfected with a different subtype than the initial virus, potentially suggesting that there are differences in the Nab responses to infection with more diverse variants.

In agreement with our functional data, computational analyses suggested that the majority of the 21 women did not develop a Nab response that could be assigned to a single known specificity with high confidence. Application of the computational method also could not definitively predict the existence of dominant responses to the more recently identified gp120-gp41 interface-targeted epitopes of Mabs PGT151 or 35O22 (S7B Fig), which is also consistent with our functional data. With the exception to QB850, the computational analysis yielded either (a) predictions that were distributed between multiple specificities without a predominant signal, or (b) a target function level, which provides a measure of confidence for the computational predictions, that was substantially higher (~25 or more) than the levels typically observed for sera with a predominant epitope specificity (~20 or less) or (c) both. Importantly, the computational analysis was consistent with the functional studies. A prior study also demonstrated that sera delineation analyses can be ~85% concordant with standard functional assays [47]. Moreover, the subtype C superinfected case whose Nab response targets V1/V2 was validated by both functional screening and computational analysis [51]. Interestingly, further mapping studies suggested that PGT-like antibodies may have constituted a minor fraction of the antibody specificity in the one individual in our cohort that was predicted with some confidence to have PGT antibodies targeting V3 glycans (QB850). In this case, removing N332 and N301 glycans from 1 of the 8 autologous clones resulted in detectable reduction in neutralization above background, but this was modest relative to the reduction we observed with the N332 supersite-specific Mab, PGT128. Furthermore, we found no evidence of escape at this epitope site, nor any other key residues from other epitopes (N88, N160, K169, N276, N611; S8A Fig).

It is common practice to identify Nab specificities by looking for loss of antibody recognition using viruses with mutations in their epitopes. Here we uncovered a seemingly underappreciated aspect of these types of epitope mapping studies, namely that these mutations also had some effect on antibody recognition by other Mabs that do not target these epitopes. To account for these non-specific effects caused by the mutation, a 3-fold cutoff was applied in experiments testing mutant viruses vs. wildtype viruses. As a result, this decreased our ability to distinguish minor fractions of the Nab response that map to these regions. However, none of the individuals tested here had neutralizing activity that was drastically affected by the loss of the residue to the same degree as the cognate Mab. Some of the non-specific effects observed with the point mutations may reflect the effects of conformational changes within the Envelope trimer. For example, it has been suggested that the CD4-binding site epitope may overlap with the V3 base epitope cluster [52], potentially explaining the reduction in VRC01’s ability to neutralize some of the N332A mutants used in this study (Fig 4). Also, removal of the N301 glycan has previously been shown to cause a global enhancement of neutralization both to pooled plasma and Mabs [53,54], which we noted when engineering this mutation into QB850.73p.E3. Although the PG9 epitope involving the glycan at position 160 is thought to be distinct from the CD4-binding site [52], it is possible that the N160 mutation affects the quaternary nature of the CD4-binding site epitope in a context-dependent manner [55], and could thus explain VRC01’s higher background with Q23 and DU156 in comparison to Q461d1.

The non-specific background observed with these assays also highlights the limitations of this method of screening. This is particularly true where the wildtype virus was not potently neutralized, since the output from this assay is a >3-fold reduction in IC50 upon introduction of a specific mutation that disrupts a known epitope. Thus, for some plasma with limited breadth and potency, it would not be possible to detect a reduction of this magnitude. Thus, we focused on the 2 women with the broadest responses in our longitudinal analysis, as in these the individuals the neutralization of the WT virus was typically potent. Moreover, since we initially set out to compare the antibody specificities in SI to single infection, it was important to use the same methods that were used in prior studies of singly infected cohorts. However, we note that our approach of utilizing both functional and computational methodologies may only detect a response that constitutes a major fraction of the breadth and potency of the response; thus, we can only conclude that these specificities are not the dominant specificity driving HIV-1 neutralization.

The identification of broad, cross-reactive Mabs from HIV-infected individuals has led to the prospect that the epitopes they target could be exploited with immunogen design [52,56]. Presentation of one or more of these epitopes with a vaccine would ideally elicit similarly potent and cross-reactive antibodies in immunized individuals, but it is unclear how such immunogens should be constructed since many immunological barriers may impede their development. For example, many of the broadly neutralizing Mabs exhibit unusual characteristics that are not typically seen in vaccine-elicited responses and in many cases, the epitopes they target are occluded on the HIV-1 Envelope [3,57,58]. It is also unclear whether potential immunogens should be designed to elicit either a focused or diverse antibody response in order to protect against infection. However, eliciting a highly diverse immune response may be favorable to providing protection against incredibly diverse HIV-1 variants in global circulation [2]. In support of this concept, previous *in vitro* studies have shown that combinations of broad Nabs that target different epitopes are required to block infection by HIV-1 transmitted variants from different subtypes [59–61]. Similarly, treating chronically-infected macaques with combinations of broadly neutralizing Mabs has also proven to be highly effective at clearing virus from circulation in the weeks following administration [62,63]. Thus, this study supports further investigations of the molecular and functional characteristics of the virus-antibody interplay in superinfected individuals, as SI may provide insight to the development of a diverse Nab response with multiple epitope specificities.

## Methods

### Ethics statement

This study was approved by the ethical review committees of the University of Nairobi, the University of Washington and the Fred Hutchinson Cancer Research Center. Written informed consent was obtained from all participants.

### Study cohort

The individuals in this study represent a subset of a prospectively followed seroincident cohort of HIV-1 negative high-risk women from Mombasa, Kenya as described [27]. Among 129 women in the Mombasa Cohort, 21 SI women were identified in a variety of studies using a combination of Sanger sequencing and 454 deep sequencing of at least 2 genome regions [28–31]. To assess Nab breadth between superinfected and singly infected individuals, 3 singly infected controls from the 129 women tested for SI in those prior studies were matched to each of the 9 recently identified SI cases based on viral subtype and sample availability ~5 years post-initial infection. Aside from sample availability, controls were assigned randomly. All women were presumably infected with HIV-1 through heterosexual contact [64], and none were using ARV therapy during follow-up for this study. Antiretroviral therapy has been provided to eligible women in the Mombasa Cohort according to Kenyan National Guidelines since March 2004.

### Cloning of Envelope variants from QB850

Single copy PCR of HIV-1 *env* from plasma obtained 73 dpi, 324 dpi, and 632 dpi was performed as previously described [65], with a few modifications. Briefly, 140ul of plasma was used to isolate virus particles by μMACS VitalVirus HIV Isolation Kit (MiltenyiBiotec) for subsequent RNA extraction with QIAamp Viral RNA Mini Spin Kit (Qiagen). HIV-1 *env* genes were amplified by nested PCR (TaqPlus Precision; Stratagene) from cDNA obtained by reverse transcription (SuperScript III; Invitrogen). PCR products were cleaned up (ExoSAP-It; Affymetrix) before direct sequencing and subsequently cloned into pCI-neo mammalian expression vectors (Promega). Sequence data from all 15 *env* clones are noted in S8A Fig and were deposited into GenBank under the following accession numbers: KT008649-KT008663. Point mutations N332A and N301A were generated by site-directed mutagenesis (QuikChange II; Agilent Technologies) and verified by sequencing.

### Pseudovirus production

Pseudoviruses were produced by co-transfecting 293T cells with cloned viral Envelopes and a full-length subtype A proviral clone with a partial deletion in envelope (Q23Δenv), as described in [27]. To assess the Nab breadth in the newly identified SI cases, the same 8-virus panel used in [27] was generated (SF162, Q461d1, Q842.d16, Q435.100M.A4, DU156.12, QC406.70M.F3, Q259.d2.26, Q769.b9), with SIV included as a negative control. Four of the viruses from this panel (Q461d1, Q842.d16, Q435.100M.A4, DU156.12) and simian immunodeficiency virus (SIV) were also used to test plasma from the 9 new cases of SI with 27 singly infected matched controls. A 21-virus panel was additionally generated to test plasma from all 21 SI cases in a sera delineation analysis. This panel included 6101.10, Bal.01, BG1168.01, CAAN.A2, DU156.12, DU422.01, JRCSF.JB, JRFL.JB, KER2018.11, PVO.04, Q168.A2, Q23.17, Q769.H6, RW020.2, THRO.18, TRJO.58, TRO.11, YU2.DG, ZA012.29, ZM106.9, and ZM55.28a.

### Neutralization assay

The TZM-bl neutralization assay was used as previously described [27]. The IC50, or reciprocal plasma dilution at which 50% of the virus was neutralized, for each plasma-virus pair was calculated using linear interpolation from the neutralization curve. A plasma sample was considered to be below the detectable limit of neutralization if the starting dilution (1:100) did not show >50% neutralization. For each virus plasma pair, two independent experiments were performed in duplicate unless indicated and a log_2_ average was calculated. For the majority of experiments, any plasma-virus pairs that demonstrated >2-fold difference in IC50 across two experiments was repeated in a third experiment and a final log_2_ average was calculated. Neutralization assays performed for the breadth and potency comparison including the new 9 SI cases and 27 matched controls was performed on a Tecan Freedom Evo 150 liquid automated system at a starting dilution of 1:100 and final dilution of 1:1600. β–Galactosidase activity for these TZM-bl neutralization assays was determined using the Gal-Screen System (Applied Biosystems).

### CD4-binding site mapping

Resurfaced core protein (RSC3) and CD4-binding site defective mutant (RSC3Δ371I) constructs were obtained though the AIDS Research and Reference Reagent Program. These proteins were produced as previously described [16], with a few modifications. Briefly, 293F cells were transfected using 293fectin (Invitrogen) and 4–5 days later supernatants were clarified by 0.45um filtration and concentrated by centrifugation in Centricon-Plus 70 filter tubes (Millipore) whilst buffer-exchanged into PBS. Proteins were purified by DEAE sepharose (GE) ion exchange chromatography, followed by His-Select Nickel (Sigma) affinity chromatography. Immulon 2HB 96-well ELISA plates (Thermo Fisher Scientific) were coated with 200ng/ml of protein in sodium bicarbonate overnight at 4°C. The plates were blocked in 10% non-fat milk with 0.3% Tween-20 in PBS and incubated with 2-fold dilutions of heat-inactivated plasma starting at a dilution of 1:100, or in some cases 1:400 to obtain an endpoint titer, before horseradish peroxidase-conjugated goat anti-human IgG antibody (BioRad) was added at a 1:3000 dilution. Plates were washed with 0.05% Tween-20 in PBS and incubations were all for 1 hour at 37°C. 1-Step Ultra TMB-ELISA substrate (Pierce Biotech) was used to develop the plate for 3 min in the dark before the reaction was stopped using 1N H_2_SO_4_ and plates were read at 450nm. Optical densities (OD) were analyzed as previously described [5]. Briefly, background-corrected OD values for the reciprocal plasma dilution greater than or equal to 0.1 were used to summarize endpoint titers. Plasma samples with endpoint titers below 1:200 were considered non-reactive. Two independent experiments were performed and an average was taken across replicates. The ratio of endpoint titers for RSC3/RSC3Δ371I were calculated, and plasma samples with a ratio >3 were further tested in a protein competition neutralization assay, as previously described [5]. Briefly, 25ul of RSC3/RSC3Δ371I protein was added at a final concentration of 25 ug/ml to an equal volume of serially diluted plasma and incubated at 37°C for 30 min before the addition of pseudovirus. The neutralization assay was then performed as described above, after a further 1 hr. incubation of protein, virus and plasma.

### Epitope mapping with point mutants

N160K, N332A, N276A, N88A, N611A and K169E point mutants were made by site-directed mutagenesis (QuikChange II, Agilent Technologies) and verified by sequencing. For consistency, IC50 values for the wildtype virus were averaged across all epitope mapping experiments and mutant IC50 values were averaged across at least two independent experiments. The ratio of wildtype IC50 to mutant virus IC50 (fold change) was then calculated as a summary measure. Calculating fold changes in IC50 per experiment and then averaging across experiments yielded similar results.

### MPER mapping

Neutralization profiles against HIV-2 (7312A) and a chimera made from HIV-2 and the HIV-1 subtype B YU-2 MPER engrafted (7312C1) were first compared [66]. Plasma samples exhibiting a greater than 3-fold change in IC50 between the HIV-2 and HIV-2/HIV-1 MPER chimera were considered candidates for further testing using a YU-2 MPER peptide (KKKNEQELLELDKWASLWNWFDITNWLWYIRKKK, GenScript) competition neutralization assay as previously described [13]. Briefly, 25ul of MPER peptide was added at a final concentration of 25 ug/ml to an equal volume of serially diluted plasma and incubated at 37°C for 30 min before the addition of pseudovirus. Neutralization assays were then performed as described above. Plasma samples exhibiting a greater than 3-fold change in IC50 with the addition of MPER peptide were considered as having evidence of MPER reactivity [13].

### Computational neutralization fingerprinting analysis

Neutralization fingerprinting analysis of plasma samples was performed as described previously [47]. Briefly, plasma neutralization data was obtained on a panel of 21 diverse HIV-1 strains (see above). The potency variability with which a given sample neutralized each of the 21 viruses was used to define the neutralization fingerprint for that sample. Plasma neutralization fingerprints were then represented as a linear combination of the neutralization fingerprints (over the same panel of viruses) of a discrete reference set of monoclonal antibodies targeting known epitopes on HIV-1 Envelope. The resulting linear coefficients (on a scale of 0 to 1) from the computational procedure were used to predict the relative prevalence of each of the reference antibody specificities in plasma neutralization. A target function level was computed as the square root of the sum of squared residuals from the computational procedure.

### Statistical analyses

Exploratory analyses assessing predictors of Nab breadth were performed using the non-parametric Mann-Whitney U test to compare across two groups and Spearman’s rank correlation to compare continuous variables. A p-value <0.05 was considered statistically significant. Breadth and potency scores were calculated and analyzed as previously described [27]. Briefly, breadth scores were analyzed using conditional Poisson regression with bootstrapped standard errors, while log transformed potency scores were analyzed using linear regression generalized estimating equation (GEE) with robust standard errors. All statistical analyses were performed with STATA statistical software (version 13, StataCorp).

## Supporting Information

S1 Fig Neutralization activity of 9 newly identified cases of SI tested against 8-virus cross-clade panel. Darker colors denote greater neutralization activity by plasma from individuals listed in the far left column, with the time point calculated as days post-initial infection (dpi), as shown in the next column. White (IC50: <100), light blue (IC50: 101–300), medium blue (IC50: 301–500), dark blue (IC50: >501). SIV is also shown as a negative control.
(TIF)

S2 Fig Competition with MPER peptides with DU156. Fold change in neutralization of the HIV-1 subtype C virus DU156 in the absence or presence of MPER peptide. >3-fold changes in IC50 are noted with red bars. 10E8, an MPER-specific Mab, is shown as a positive control and VRC01, a CD4-binding site-specific Mab, is shown as a negative control. *Actual fold change is >1.56 since 10E8 potently neutralizes DU156 (IC50: <0.33 ug/ml; [13] published IC50 = 0.007 ug/ml). N/A denotes a time point not tested in these experiments. dpSI, days post-SI.
(TIF)

S3 Fig The ability of RSC3 and RSC3Δ371I proteins to compete for CD4-binding site-specific antibodies in a neutralization assay. Mabs VRC01, a CD4-binding site specific Nab, and PG9, a V1/V2 glycan-specific Nab, tested against Q461d1, DU156, and Q23 with the addition of RSC3 wildtype or mutant proteins. Fold change in IC50 comparing neutralization in the absence or presence of either protein is listed, with fold changes >3 highlighted in red.
(TIF)

S4 Fig Inhibition of neutralization by point mutations that disrupt V1/V2 and V3 glycan-specific Nab recognition. IC50 values for each set of WT and mutant virus pairs are shown in both panels for all 21 cases of SI, with colors denoting the different mutated residues, corresponding to specific epitopes targeted on Envelope by Mabs listed above for N160K and N332A/N334A/N301A mutations. N/A denotes plasma samples that were unable to neutralize the wildtype virus.
(TIF)

S5 Fig Inhibition of neutralization by point mutations that disrupt epitopes outside the 4 main regions. IC50 values for each set of WT and mutant virus pairs are shown for all 21 cases of SI, with colors denoting the different mutated residues, corresponding to specific epitopes targeted on Envelope by Mabs listed above for the 4 different mutations. N/A denotes plasma samples that were unable to neutralize the wildtype virus.
(TIF)

S6 Fig Longitudinal analysis of epitope targets in QB850 and QA013 plasma. IC50 values for each set of WT and mutant virus pairs are shown, with colors denoting the different mutated residues, corresponding to specific epitopes targeted on Envelope by Mabs listed above for the 6 different mutations. N/A denotes plasma samples that were unable to neutralize the wildtype virus.
(TIF)

S7 Fig Computational analysis comparing neutralization fingerprints of plasma from 21 SI cases to Mabs with known epitope specificities. The result from the serum neutralization fingerprinting analysis for 10 specificities on all 21 cases of SI is shown (A), with cases sorted according to Table 1. The data displayed in the center panel are shown as the relative scores for each Mab type (columns) against each plasma (rows) on a scale of 0–1, with a higher number and darker coloring denoting a greater likelihood that a particular specificity is present in plasma. Percent of viruses neutralized shows is based the 21-virus panel used in the analysis. The results for two individuals (QD151 and QB008) are grayed out to show that <25% of viruses were neutralized and delineation analyses were not assessed. Target function level is a measure of confidence for the predicted scores, with a lower score and lighter shading denoting higher confidence. The second analysis including neutralization fingerprints from the two recently identified Mabs that target the gp120-gp41 interface (PGT151 and 35O22) is also shown for all 21 cases (B), and ordered with the first 12 SI cases before the 9 new cases.
(TIF)

S8 Fig Sequence alignment of Envelope variants cloned from QB850 and their neutralization profiles against autologous plasma. Alignment of the initial and SI recombinant Envelopes isolated from QB850 plasma collected 73 dpi, 324 dpi, and 632 dpi (A). Red names denote the initial Subtype A Envelope, while blue denotes SI recombinants. Clone names are listed as the time point, followed by a “p” to denote it was isolated from plasma and then the PCR (letter) and colony isolated (#). (B) Longitudinal autologous neutralization of QB850 initial and superinfecting recombinant viruses from 73 dpi (D), 324 dpi (C), and 632 dpi (D). White (IC50: <100), light blue (IC50: 101–300), medium blue (IC50: 301–500), dark blue (IC50: >501). dpi, days post-initial infection; ND, not done.
(TIF)
