# Supplementary figures and images for: The Broad Neutralizing Antibody Responses after HIV-1 Superinfection Are Not Dominated by Antibodies Directed to Epitopes Common in Single Infection
Source: PLoS Pathog. 2015 Jul 9;11(7):e1004973. doi: 10.1371/journal.ppat.1004973 (PMC4497680; doi:10.1371/journal.ppat.1004973)

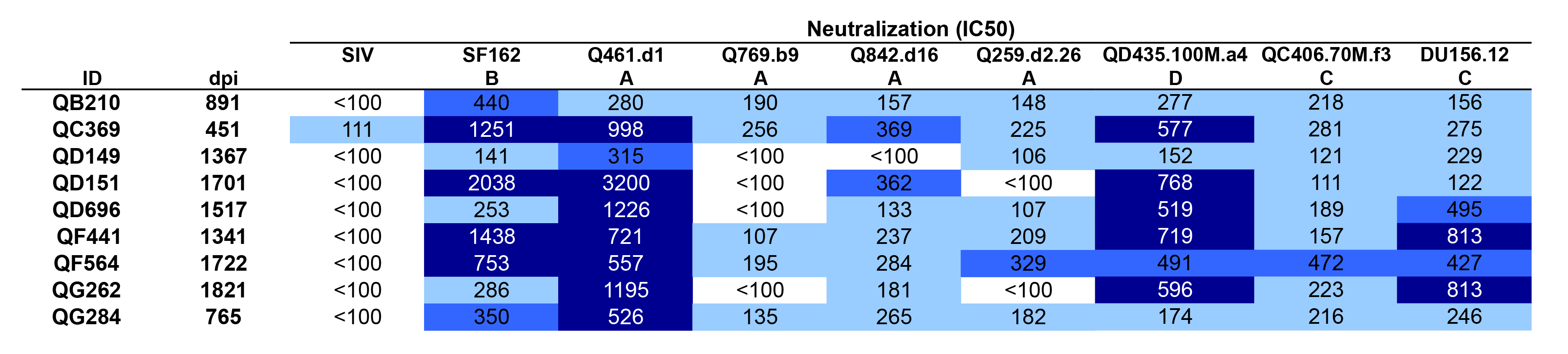

Supplement: S1 Fig — Darker colors denote greater neutralization activity by plasma from individuals listed in the far left column, with the time point calculated as days post-initial infection (dpi), as shown in the next column. White (IC50: <100), light blue (IC50: 101–300), medium blue (IC50: 301–500), dark blue (IC50: >501). SIV is also shown as a negative control. (TIF) [file ppat.1004973.s001.tif]

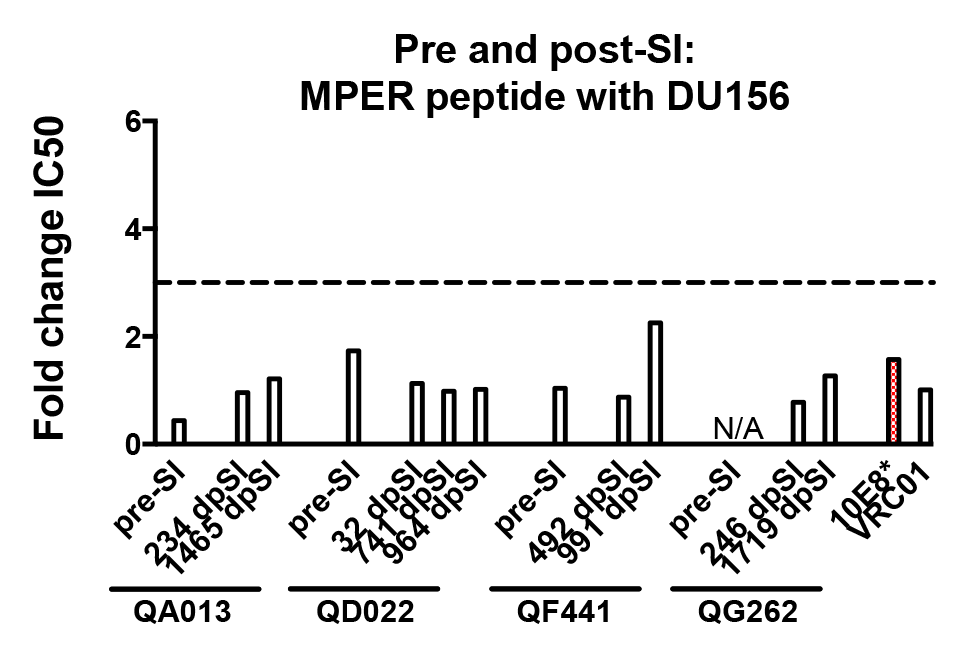

Supplement: S2 Fig — Fold change in neutralization of the HIV-1 subtype C virus DU156 in the absence or presence of MPER peptide. >3-fold changes in IC50 are noted with red bars. 10E8, an MPER-specific Mab, is shown as a positive control and VRC01, a CD4-binding site-specific Mab, is shown as a negative control. *Actual fold change is >1.56 since 10E8 potently neutralizes DU156 (IC50: <0.33 ug/ml; [13] published IC50 = 0.007 ug/ml). N/A denotes a time point not tested in these experiments. dpSI, days post-SI. (TIF) [file ppat.1004973.s002.tif]

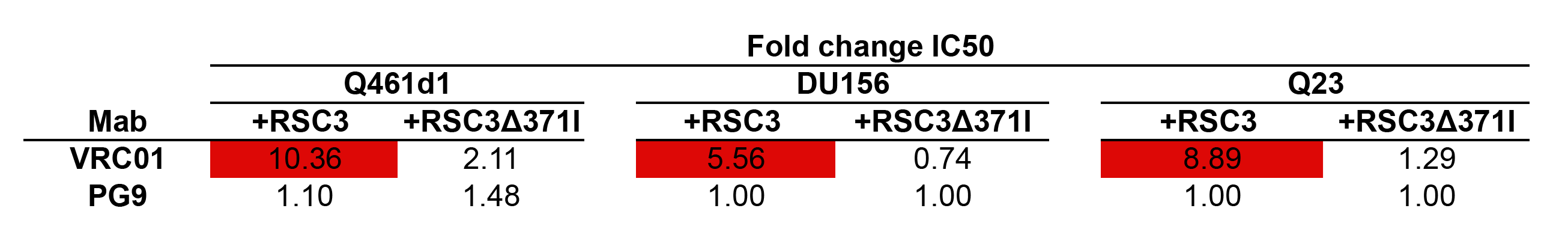

Supplement: S3 Fig — Mabs VRC01, a CD4-binding site specific Nab, and PG9, a V1/V2 glycan-specific Nab, tested against Q461d1, DU156, and Q23 with the addition of RSC3 wildtype or mutant proteins. Fold change in IC50 comparing neutralization in the absence or presence of either protein is listed, with fold changes >3 highlighted in red. (TIF) [file ppat.1004973.s003.tif]

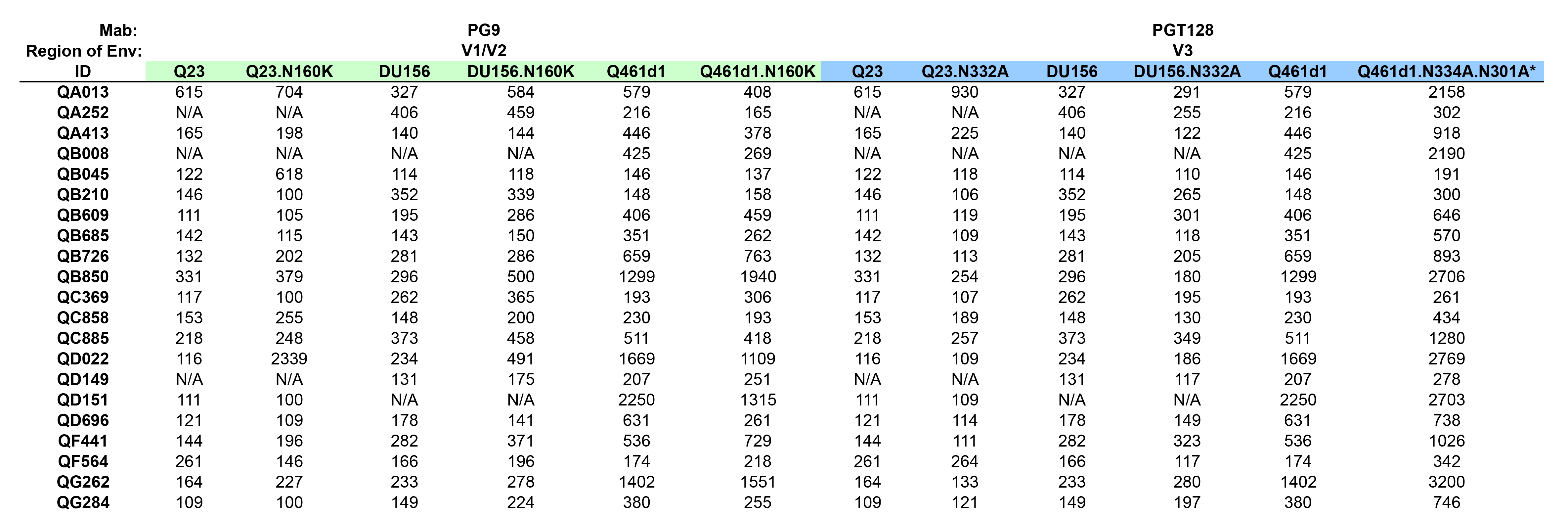

Supplement: S4 Fig — IC50 values for each set of WT and mutant virus pairs are shown in both panels for all 21 cases of SI, with colors denoting the different mutated residues, corresponding to specific epitopes targeted on Envelope by Mabs listed above for N160K and N332A/N334A/N301A mutations. N/A denotes plasma samples that were unable to neutralize the wildtype virus. (TIF) [file ppat.1004973.s004.tif]

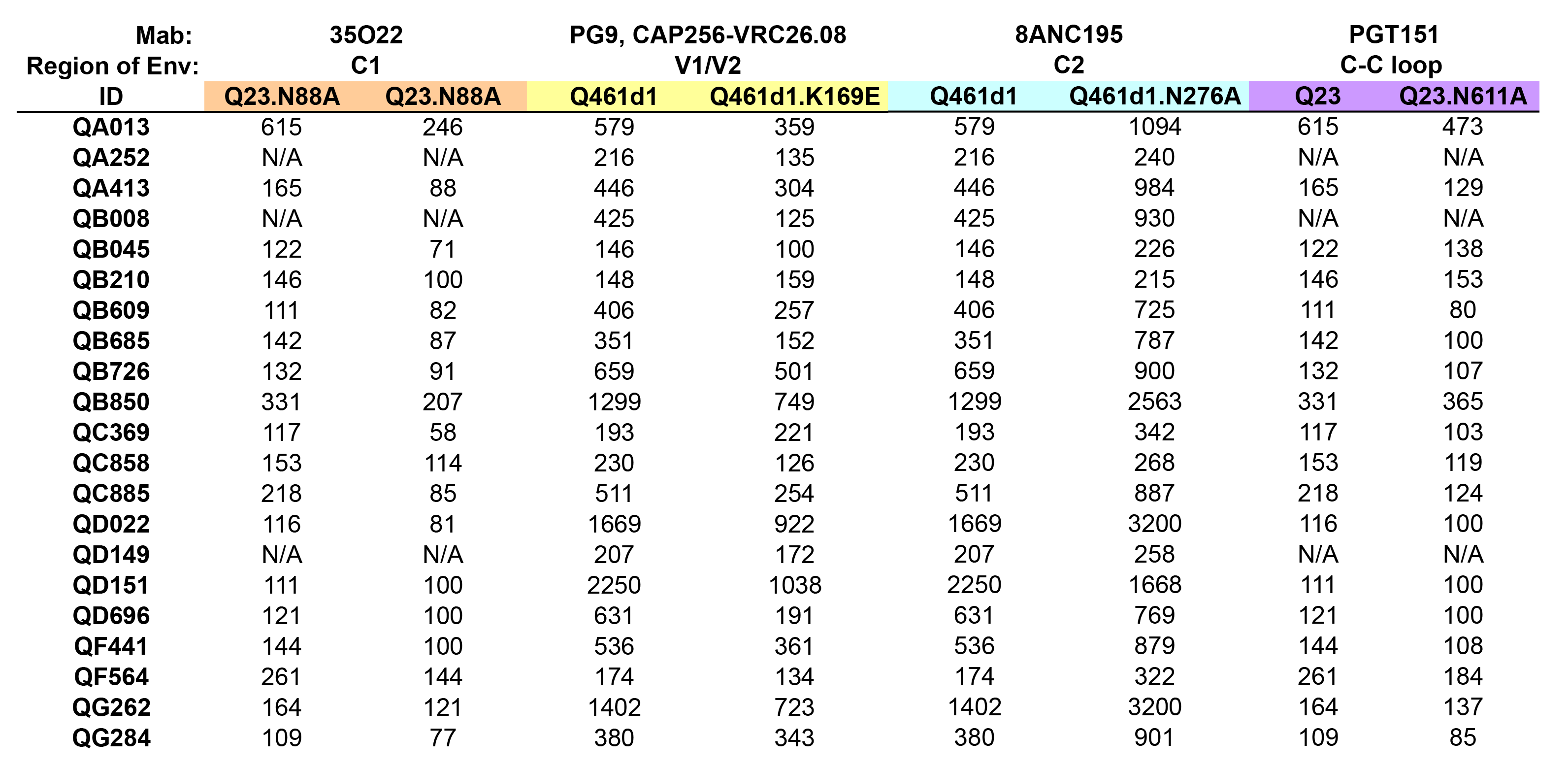

Supplement: S5 Fig — IC50 values for each set of WT and mutant virus pairs are shown for all 21 cases of SI, with colors denoting the different mutated residues, corresponding to specific epitopes targeted on Envelope by Mabs listed above for the 4 different mutations. N/A denotes plasma samples that were unable to neutralize the wildtype virus. (TIF) [file ppat.1004973.s005.tif]

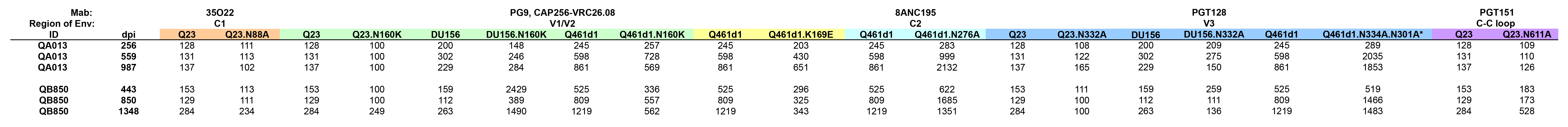

Supplement: S6 Fig — IC50 values for each set of WT and mutant virus pairs are shown, with colors denoting the different mutated residues, corresponding to specific epitopes targeted on Envelope by Mabs listed above for the 6 different mutations. N/A denotes plasma samples that were unable to neutralize the wildtype virus. (TIF) [file ppat.1004973.s006.tif]

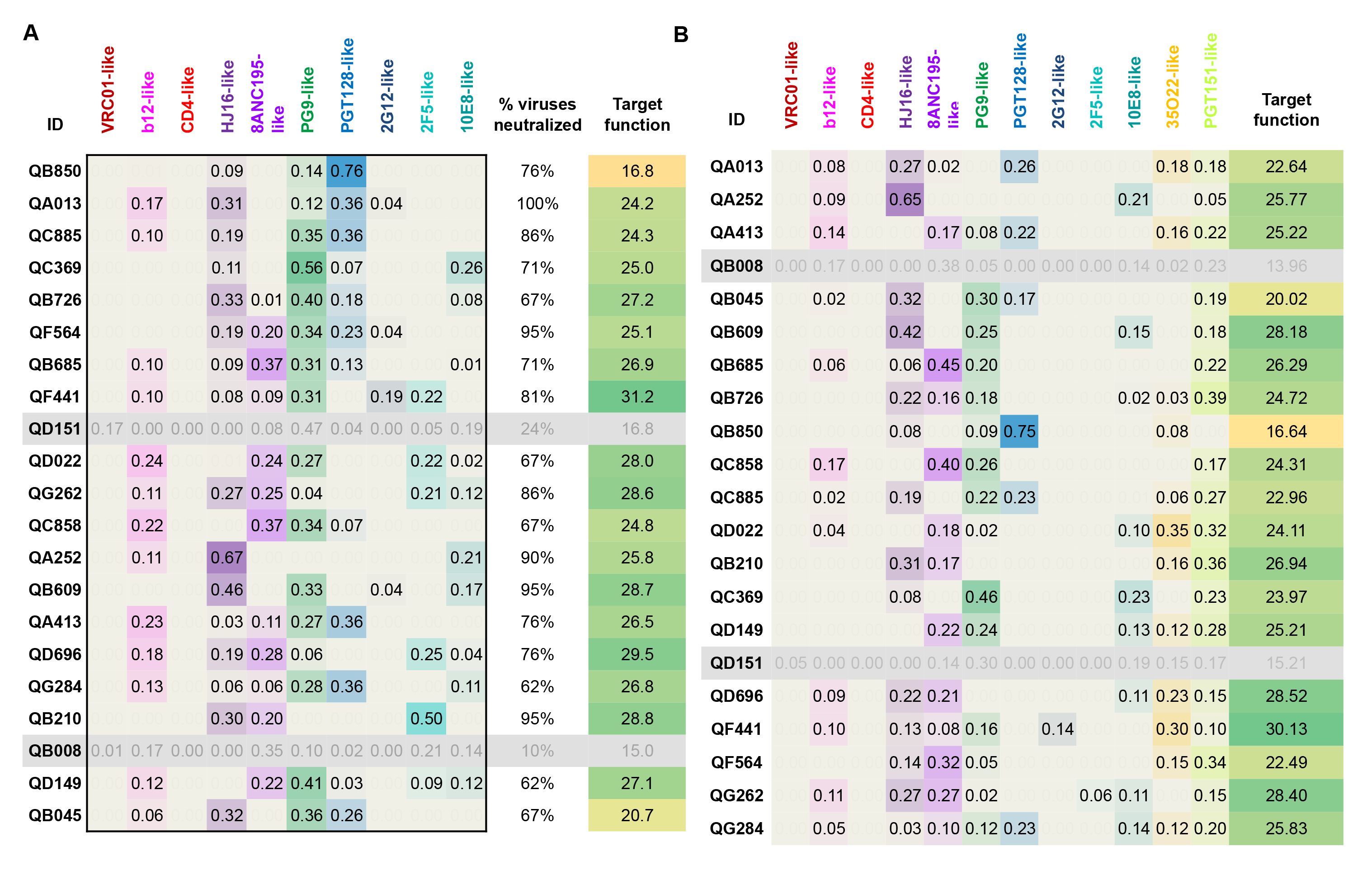

Supplement: S7 Fig — The result from the serum neutralization fingerprinting analysis for 10 specificities on all 21 cases of SI is shown (A), with cases sorted according to Table 1. The data displayed in the center panel are shown as the relative scores for each Mab type (columns) against each plasma (rows) on a scale of 0–1, with a higher number and darker coloring denoting a greater likelihood that a particular specificity is present in plasma. Percent of viruses neutralized shows is based the 21-virus panel used in the analysis. The results for two individuals (QD151 and QB008) are grayed out to show that <25% of viruses were neutralized and delineation analyses were not assessed. Target function level is a measure of confidence for the predicted scores, with a lower score and lighter shading denoting higher confidence. The second analysis including neutralization fingerprints from the two recently identified Mabs that target the gp120-gp41 interface (PGT151 and 35O22) is also shown for all 21 cases (B), and ordered with the first 12 SI cases before the 9 new cases. (TIF) [file ppat.1004973.s007.tif]

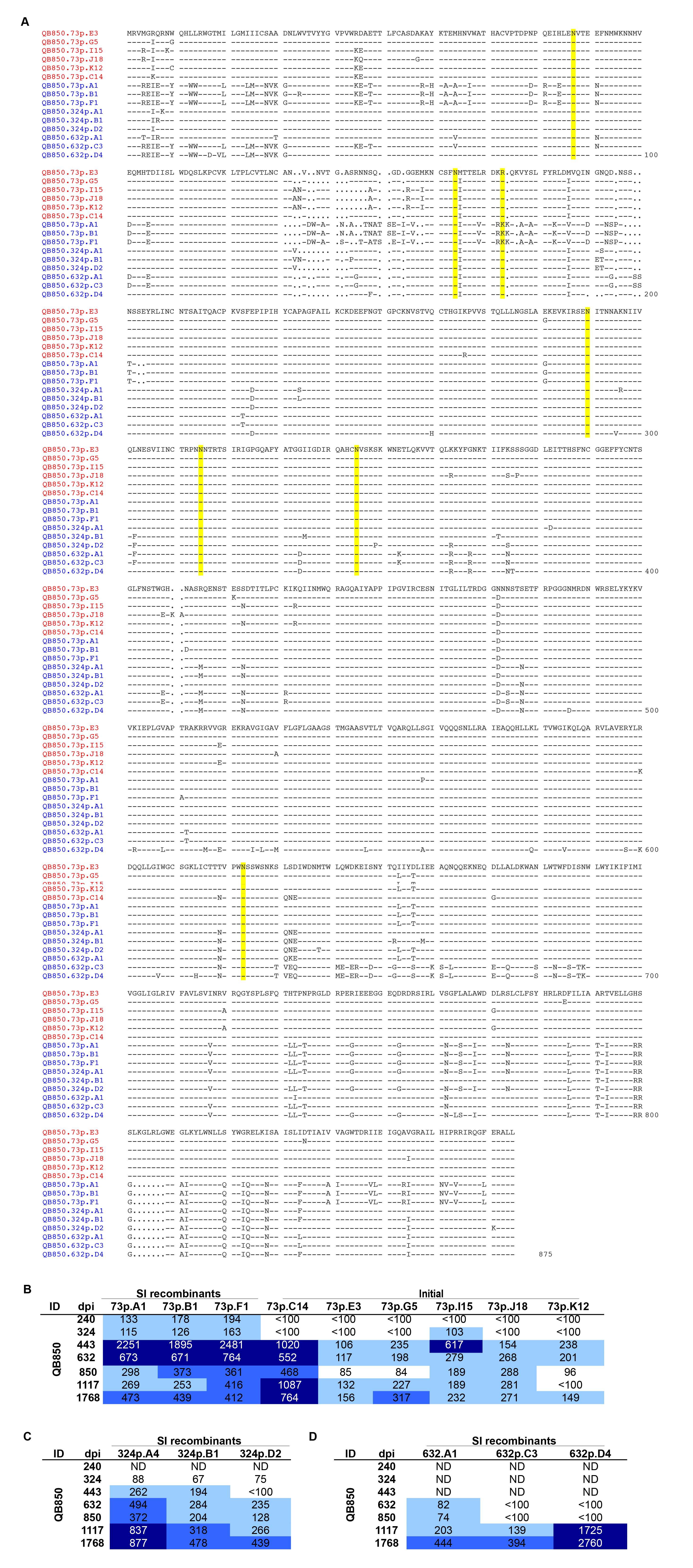

Supplement: S8 Fig — Alignment of the initial and SI recombinant Envelopes isolated from QB850 plasma collected 73 dpi, 324 dpi, and 632 dpi (A). Red names denote the initial Subtype A Envelope, while blue denotes SI recombinants. Clone names are listed as the time point, followed by a “p” to denote it was isolated from plasma and then the PCR (letter) and colony isolated (#). (B) Longitudinal autologous neutralization of QB850 initial and superinfecting recombinant viruses from 73 dpi (D), 324 dpi (C), and 632 dpi (D). White (IC50: <100), light blue (IC50: 101–300), medium blue (IC50: 301–500), dark blue (IC50: >501). dpi, days post-initial infection; ND, not done. (TIF) [file ppat.1004973.s008.tif]
